# Comparison of primary repair and patch plasty procedure on the P wave in adult atrial septal defect closure

**DOI:** 10.5830/CVJA-2016-013

**Published:** 2016

**Authors:** Alper Ucak, Veysel Temizkan, Murat Ugur, Arif Selcuk, Ahmet Turan Yilmaz, Ahmet Erturk Yedekci, Omer Uz

**Affiliations:** Department of Cardiovascular Surgery, GATA Haydarpasa Training Hospital, Istanbul, Turkey; Department of Cardiovascular Surgery, GATA Haydarpasa Training Hospital, Istanbul, Turkey; Department of Cardiovascular Surgery, GATA Haydarpasa Training Hospital, Istanbul, Turkey; Department of Cardiovascular Surgery, GATA Haydarpasa Training Hospital, Istanbul, Turkey; Department of Cardiovascular Surgery, GATA Haydarpasa Training Hospital, Istanbul, Turkey; Department of Anesthesiology, Kyrenia Military Hospital, Cyprus; Department of Cardiology, GATA Haydarpasa Training Hospital, Istanbul, Turkey

**Keywords:** atrial septal defect, P-wave analysis, arrhythmia

## Abstract

**Introduction:**

In this study we compared the effects of two different surgical procedures for closure of adult atrial septal defect (ASD) on postoperative P-wave changes.

**Methods:**

Patients who underwent cardiac surgery for secundum type ASD closure were evaluated retrospectively. Seventy-two patients with primary repair of ASD and 29 patients with pericardial patch plasty repair were compared according to P_max_, P_min_ and P-wave dispersions (P_d_).

**Results:**

In each group, the increases in postoperative maximum P-wave duration (P_max_) and minimum P-wave duration (P_min_) were statistically significant. There was no statistically significant difference between post- and pre-operative P_d_ values. In the comparison between group 1 and group 2 in terms of postoperative P-wave changes (P_max_, P_min_, P_d_) there was no statistically significant difference.

**Conclusion:**

Comparing patch plasty and primary repair for the surgical closure of ASD in the early to mid-postoperative period, no difference was found and both surgical procedures can be performed in adult ASDs.

## Introduction

Atrial septal defect (ASD) is one of the most common congenital heart defects in adulthood. It can be repaired percutaneously or surgically, depending on the defect size. Primary surgical repair or patch plasty (closure with patch) are the two surgical ASD closure techniques used depending on diameter of the defect.

Atrial arrhythmias may develop in repaired and unrepaired ASD patients. The inter-atrial conduction pathway may influence this, and postoperative arrhythmias may develop due to increased size of the right atrium in unrepaired patients, or tension in the suture line in the postoperative period.^1^ Prolonged maximum P-wave duration (P_max_) and increased P dispersion (P_d_) may be pioneer indicators of disturbance of the inter-atrial conduction pathway and atrial fibrillation in ASD patients.^2^

Many different studies have been carried out for P-wave changes following ASD closure surgeries. These studies evaluated the effects of percutaneous or surgical closure techniques on the P wave after defect repair.^3^-^6^ In the literature, there is no study investigating the effects of surgical ASD closure techniques on the P wave in adults. In our study, we retrospectively investigated the electrocardiograms of adult patients who underwent surgical closure of ASDs and evaluated the effects of both primary closure and pericardial patch plasty techniques on the P wave.

## Methods

The study design was approved by the institutional review board. Patients who underwent surgical ostium secundum type ASD closure between the years 2004 and 2014 in the cardiovascular surgery clinic of the GATA Haydarpasa Training Hospital were included in the study. Patients with primum ASD and patients with cardiac pathologies requiring additional surgical treatment were excluded from the study.

Twelve-lead surface electrocardiograms were collected from patients’ records who underwent surgical ostium secundum type ASD closure. The 101 patients were divided into two groups (Table 1) according to their closure procedure; primary surgical repair (group 1) and pericardial patch plasty (group 2).

**Table 1:** Comparison of pre-operative patients’ characteristics

*Demographics*	*Group 1 (n = 72)*	*Group 2 (n = 29)*
Age (years)	21.8 ± 2.4	22.3 ± 3.7
BMI (kg/m^2^)	25.2 ± 1.2	24.7 ± 1.3
Diameter of defect (mm)	20.6 ± 8.5	23.9 ± 10.3
Qp/Qs	2.0 ± 0.7	2.3 ± 0.9
EF (%)	60.9 ± 6.2	64.3 ± 5.2
PASB (mmHg)	30 ± 10.7	32.3 ± 11.8
≤ Mild tricuspid regurgitation (n)	5	1
≤ Mild pulmonary regurgitation (n)	1	0
Anomalous pulmonary venous return (n)	0	1
≤ Mild pulmonary stenosis (n)	2	1
≤ Mild aortic regurgitation (n)	1	0

BMI: body mass index, EF: ejection fraction, PASB: pulmonary artery systolic pressure.

Seventy-two patients without increased atrial stretch and with a small- to medium-sized defect diameter were evaluated as suitable for primary surgical repair (group 1) and they underwent primary surgical repair for ASD closure. Twenty-nine patients with a larger defect diameter and/or patients with accompanying sinus venosus type ASD (group 2) underwent the patch plasty technique for ASD closure. The demographic data of the patients in each group and their pre- and postoperative five- to seven-day and three-month 12-lead surface electrocardiogram P waves were compared.

All surgeries were carried out under general anaesthesia. A median sternotomy, mini-thoracotomy and mini-sternotomy were performed in 69, 14 and 18 patients, respectively (Table 2). Cardiopulmonary bypass was achieved by cannulation of the aorta and double venous cannulation in the right atrium following median sternotomy, where femoral arterial cannulation and femoral vein to selected superior vena cava cannulation were achieved following mini-thoracotomy/mini-sternotomy.

**Table 2:** Comparison of surgical data

*Surgery*	*Group 1 (n = 72)*	*Group 2 (n = 29)*
Median sternotomy (n)	44	25
Mini-sternotomy (n)	17	1
Mini-thoracotomy (n)	11	3
CPB time (min)	38.5 ± 12.2	42.4 ± 16.6
Cross-clamp time (min)	20.3 ± 7.6	23.3 ± 10.6
Revision (n)	1	0
Hospital stay (days)	6.2 ± 1.4	6.4 ± 1.4

CPB: cardiopulmonary bypass.

Cardiopulmonary bypass was established and following cardioplegic arrrest, 32°C blood–body temperature was provided. Following right atriotomy, the defect was evaluated in the presence of pre-operative echocardiographic findings. In group 1, the defect was closed with a primary continuous suture technique with 4/0 prolene. In group 2, the defect was closed by means of a fresh autologous pericardial patch using a continuous suture technique with 4/0 prolene.

Pre-operative, fifth- and seventh-day postoperative and threemonth 12-lead surface electrocardiograms were provided frompatients’ records, which were obtained at a paper speed of 50 mm/s with 1-mV/cm standardisation. Electrocardiograms were scanned for evaluation of the P waves. Electrocardiograms exhibiting P waves at least nine in 12 derivations were analysed for the existing P waves.^4^

Measuring the length of the P waves using Photoshop® (Adobe), the longest P wave was denoted as P_max_ whereas the shortest P wave was P_min_. The difference between P_max_ and P_min_ was the P-wave dispersion (P_d_) (P_d_ = P_max_ – P_min_).

## Statistical analysis

The 5.0 version of the GraphPad Prism program was used for statistical analysis. Data are shown as mean ± standard deviation. Postoperative P-wave changes of the patients in group 1 and 2 were compared with pre-operative values and the differences were evaluated. Continuous variables were compared using Mann–Whitney *U*- and Student’s *t*-tests. A p-value ≤ 0.05 was considered statistically significant.

## Results

Pre-operative demographic characteristics of the two groups were similar. Demographic characteristics and transthoracic\ echocardiographic data of our patients are shown in Table 1. All of the patients were in sinus rhythm in the pre-operative period. Arrhythmia was not observed in the postoperative follow up and there was no need for pace implementation. The patients were discharged 6.2 ± 1.4 days postoperatively.

In group 1, compared to the pre-operative period, P_max_ was significantly increased in the five to seven days postoperatively, and P_max_ was still significantly longer three months after the procedure (Table 3). P_max_ was also increased in the postoperative period in group 2 but this change gained statistical significance at three months following the procedure (Table 4). In the evaluation of P_min_, compared to the pre-operative period, P_min_ was significantly increased in the five to seven days postoperatively and three months after the procedure in both groups (Table 3, Table 4).

**Table 3:** P-wave changes in primary repair procedure for ASD closure

	*Pre-operative*	*Postoperative 5th day*	*Postoperative 3rd month*	*P_1_*	*P_2_*
P_max_	205.9 ± 29.4	220.6 ± 31.5	231.1 ± 39.4	0.0033	0.0001
P_min_	108.1 ± 29.4	121.2 ± 32.7	129.5 ± 36.9	0.0162	0.0003
P_d_	97.2 ± 33.1	98.8 ± 35.9	101.7 ± 42.2	0.7011	0.4432

p_1_: comparison of the pre-operative period and the fifth day postoperatively; p_2_: comparison of the pre-operative period and the third month postoperatively.

**Table 4:** P-wave changes in pericardial patch plasty procedure for ASD closure

	*Pre-operative*	*Postoperative 5th day*	*Postoperative 3rd month*	*P_1_*	*P_2_*
P_max_	219.4 ± 37.7	236.1 ± 39.4	254.3 ± 51.1	0.1092	0.0089
P_min_	107.1 ± 28.7	120.3 ± 27.7	132.8 ± 35.6	0.06	0.043
P_d_	110.6 ± 43.6	115.9 ± 39.9	121.7 ± 47.9	0.5659	0.2796

p1: comparison of the pre-operative period and the fifth day postoperatively; p2: comparison of the pre-operative period and the third month postoperatively.

Evaluation of the P-wave dispersion revealed that in group 1, compared to the pre-operative period, no significant difference was found in the five to seven days postoperatively and three months after the procedure. Similarly, in group 2, compared to the pre-operative period, no significant difference was found in any postoperative follow-up periods. No statistically significant difference was found in a comparison of group 1 and 2, both pre-operatively and at the postoperative follow up in terms of P-wave analysis (P_max_, P_min_ and P_d_) (Table 5).

**Table 5:** Comparison of primary repair procedure and pericardial patch plasty procedure for ASD closure

	*Primary repair*	*Patch plasty*	*p-value*
Pre-operative			
P_max_	205.9 ± 29.4	219.4 ± 37.7	0.1112
P_min_	108.1 ± 29.4	108.8 ± 28.7	0.9436
P_d_	97.2 ± 33.1	110.6 ± 43.6	0.1742
Postoperative 5th day			
P_max_	220.6 ± 31.5	236.1 ± 39.4	0.5805
P_min_	121.2 ± 32.7	120.3 ± 27.7	0.983
P_d_	98.8 ± 35.9	115.9 ± 39.9	0.0929
Postoperative 3rd month			
P_max_	231.1 ± 39.4	254.3 ± 51.1	0.0639
P_min_	129.5 ± 36.9	132.7 ± 35.6	0.6696
P_d_	101.7 ± 42.2	121.7 ± 47.9	0.0711

## Discussion

The presence of ASD causes volume overload and increased stretch induces right heart dilatation and dysfunction. In these patients, P_max_ and P_d_ extension develops as a result of prolongation of the atrial depolarisation time.^2^,^4^,^7^ The prolongation of P_d_ and P_max_ reflect non-homogeneous and discontinuous sinus stimulation and may be predictors of atrial fibrillation.^2^

In the literature, P-wave changes have been compared in repaired and unrepaired ASDs, or in repaired ASDs by surgical or percutaneous means. There is no study comparing the effects of two different surgical techniques on the P wave. In our study, two different surgical ASD closure techniques were compared in terms of P-wave analyses using primary repair and pericardial patch plasty techniques.

The incidence of dysrhythmia increases with increasing age in patients with unrepaired ASD.^8^ Additionally, the risk of arrhythmia increases due to right atrial volume overload and atrial remodelling related to atrial hypertrophy.3 One of the indications of ASD closure is to avoid arrhythmia, which increases the risk of morbidity and mortality.^2^,^7^ The risk of atrial arrhythmia decreases with recovery of right atrial dilatation and electrophysiological changes after surgical closure of the ASD.^9^

In our study, we compared the effects of two surgical techniques on atrial conduction pathways and remodelling caused by inflammatory fibrosis, by analysis of the P wave. In both primary repair and patch plasty groups, P_max_ and P_min_ values increased but P_d_ values did not change. Increase in P_max_ and P_min_ values in the third month may have been related to early remodelling, however because of the unchanged P_d_, we believe that the risk of arrhythmia will not increase in the long term.

In the postoperative period, the P_d_ value decreased in patients who had regressed right atrial dilatation. However, P_max_ and P_d_ were longer in patients with persistent atrial dilatation.^10^ From the results of Fang et al.,^10^ longer P_d_values in patients with permanent atrial dilatation support the notion that P_d_may be used as a non-invasive parameter for prediction of atrial arrhythmia.

Our findings showed that the risk of atrial arrhythmia was similar in both primary closure and patch plasty techniques, since there was no difference between P_d_ values. By contrast, Thilen et al.^11^ declared that increased P-wave duration did not decrease with surgical repair in older patients and it was not related to atrial dilatation. In haemodynamically significant ASD, the increase in P-wave duration may depend on regional damage of the atrial conduction pathways rather than atrial enlargement.^12^,^13^

In our study, we were able to obtain three-month follow-up records of the patients. During the follow up, although P_max_values had increased, P_d_ values did not change and there was no arrhythmia. From our results, we believe that in repair of ASD in adults, the P_d_value could be a more meaningful predictor for the risk of arrhythmia.

The age of the patient at ASD closure may be a risk factor for the development of arrhythmia in the follow-up period.^11^,^14^ ASD repair in childhood prevents permanent changes in the atrial myocardium and regresses P_max_ and P_d_, thus decreasing the risk for atrial fibrillation in older patients.^2^,^15^ In adult patients, the chance of returning to normal atrial size is lower with ASD repair. Repair of ASD after 25 years of age is a risk factor for the development of atrial fibrillation in the long term.^14^,^16^

In our study we investigated 101 patients who had had a diagnosis of ASD. Ninety of them were younger than 25 years and their mean age was 21.9 ± 2.8 years. None of the patients had arrhythmia before the surgery and none had atrial arrhythmia during the three-month follow-up period. In our patients, the P_d_ value did not change after surgical repair and the rate of returning normal atrial size may have been higher since most of our patients were young adults.

The ASD closure technique did not affect the P wave in a comparison of different percutaneous techniques, or surgical repair and percutaneous techniques in previous studies.^3^,^11^ Javadzadegan et al.^3^ suggested that in both percutaneous ASD closure and surgical treatment, the P-wave duration was decreased at six months’ follow up and this decrease was not related to the defect size. In comparison, surgical and transcatheter ASD closure, Baspınar et al.^7^ declared that in the surgical group, decrease in the P_d_ was more meaningful in the early period.

We compared primary closure and patch plasty techniques as two different surgical techniques, to analyse the effects on the P wave, and there were no differences between the two surgical techniques. When considering the anatomy of the right atrial electrophysiological conduction pathway, there are no significant conduction pathways into the atrial septum. We believe that different surgical closure techniques do not affect the P_d_ value or cause changes.

In long-term follow up after ASD closure, increase in Pd values could be a sign of atrial arrhythmia.^2^ In ASD repair of young adults, there was no increase in Pd values following ASD closure. Neither primary repair nor patch plasty techniques had any effect on the P-wave length or dispersion. Both surgical techniques can therefore be performed, depending on the defect anatomy and size.

There are limitations to this study. The majority of patients were young and they were in the second and third decades oftheir lives. After three months’ postoperative period, we had limited access to long-term follow-up records, because they were carried out in their homelands. We need long-term results to assess whether the increased P_max_ and P_d_ values in the first three months continue in the long term and whether these changes led to arrhythmias in the follow-up period.

## Conclusion

In this study we compared patch plasty and primary repair for the surgical closure of ASD in the early to mid-postoperative period. No differences were found between the methods in terms of postoperative P-wave changes, and we concluded that both surgical procedures can be performed in adult ASDs.
